# Buruli Ulcer in Gabon, 2001–2010

**DOI:** 10.3201/eid1807.110613

**Published:** 2012-07

**Authors:** Ulysse Ateba Ngoa, Gregoire K. Adzoda, Bayonne Manou Louis, Ayola Akim Adegnika, Bertrand Lell

**Affiliations:** Albert Schweitzer Hospital, Lambaréné, Gabon (U. Ateba Ngoa, G.K. Adzoda, A.A. Adegnika, B. Lell);; Ministry of Health, Libreville, Gabon (B.M. Louis);; University of Tübingen, Tübingen, Germany (U. Ateba Ngoa, A.A. Adegnika, B. Lell);; and Leiden University Medical Center, Leiden, the Netherlands (A.A. Adegnika)

**Keywords:** Buruli ulcer, Mycobacterium ulcerans, Gabon, Africa, mycobacteria, bacteria

**To the Editor**: Worldwide, Buruli ulcer is the third most common mycobacterial infection, following only tuberculosis and leprosy (*1**,**2*). It has been identified in 30 countries, including 12 African countries (*1**–**3*). For Gabon, the first report of a case consistent with Buruli ulcer was published in 1961 (*4*). The patient was a European woman who sought care at a hospital in Lambaréné for a painless upper arm nodule, which evolved into a plaque and then an extensive ulcer. The only other Buruli ulcer reports available for Gabon are a case report from 1968 and a case-series report from 1986 (*5**,**6*). We report data for Buruli ulcer in this sub-Saharan African country for 2001–2010, including prevalence within a hospital population and clinical presentation of the cases. These data can be used to assess long-term developments in the number of cases of Buruli ulcer in this region.

In Gabon, the major focus of Buruli ulcer is the area around Lambaréné (population ≈25,000), the capital of Moyen Ogooué Province (population ≈35,000). It is located near the equator in the central African rainforest. Lambaréné lies near the confluence of 2 major rivers, Ogooué and Ngounié, and is the starting point for one of the largest river deltas in Africa. Numerous lakes are present throughout the region.

The Albert Schweitzer Hospital in Lambaréné serves the entire province. At this hospital, Buruli ulcer is diagnosed on the basis of clinical presentation. In addition, tissue samples are sent to the Prince Leopold Institute of Tropical Medicine in Belgium for PCR analysis. All cases are treated surgically, and since 2006, patients have received rifampin and streptomycin as well. Since 2007, patient information has been recorded on a BU-02 form, designed by the World Health Organization to register and report cases of Buruli ulcer (*1*).

We reviewed cases of Buruli ulcer at the Albert Schweitzer Hospital. We checked the hospital registry and patient records from 2001 through 2010 to identify probable cases of Buruli ulcer on the basis of clinical appearance and response to treatment. We also gathered information from BU-02 forms from 2007 through 2010.

During 2001–2010, the number of patients admitted to surgical wards because of suspected Buruli ulcer ranged from 5 to 40 per year (average 25 patients/year) (Figure). Despite moderate variability from year to year, the number of cases over the years increased (χ^2^ for trend, p = 0.003), which could be associated with increased awareness of the disease. The variability was not caused by changes in the number of patients hospitalized.

**Figure:**
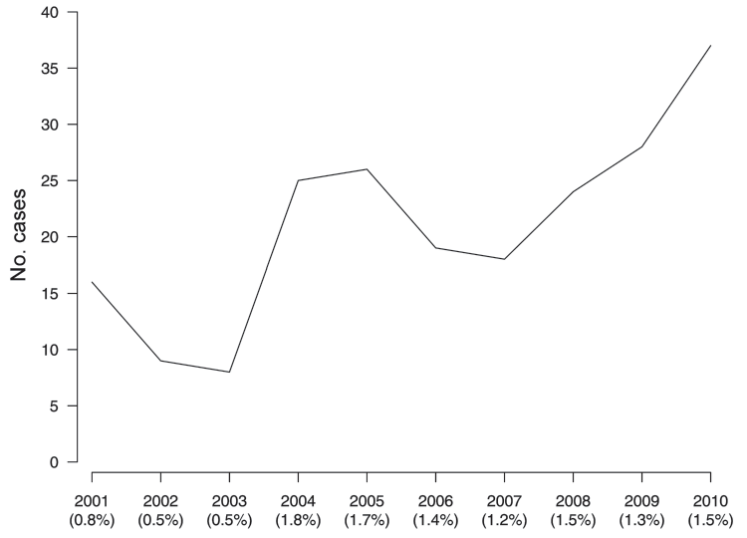
Number (line) and prevalence (in parentheses) of Buruli ulcer cases, Gabon, 2001–2010.

During 2007–2010, detailed clinical information from BU-02 forms was available for 77 patients. PCR results were available for 57 patients and confirmed the diagnosis for 39. Patient ages ranged from 2 to 72 years; 40 (52%) patients were <15 years of age. The male/female ratio was 0.83. For 44 (57%) of the patients, Buruli ulcer was a new diagnosis. In addition, 56 (73%) patients had an ulcerative lesion, and 21 (37%) of these had lesions >5 cm. The lesions were located on the lower arm for 41 (53%) patients, upper arm for 28 (36%) patients, chest and/or back for 7 (9%) patients, and perineal region for 1 (1%) patient.

Depending on the type of lesion, the length of hospitalization ranged from 1 to 352 days (median 31 days). The longest hospitalization was almost 1 year; the patient was a child who had severe lesions and lived in conditions in which adequate wound care and follow-up after hospital discharge were unlikely.

In Gabon, the available data on Buruli ulcer come mainly from surgical wards in areas where prevalence is high. A national survey of hospital registration data in 2005 detected 3 cases in Ngounie Province in southern Gabon and 5 cases in Woleu-Ntem Province in northern Gabon. All cases are thought to have been acquired locally, thus establishing the existence of 2 previously unknown foci (U. Ateba Ngoa et al., unpub. data).

Buruli ulcer has a strong economic effect on the community and health facilities. For example, in 2010, management of the disease at the Albert Schweitzer Hospital cost an estimated 554–1,660 euros per person, not including drug costs (*7*). In 2009, African countries where Buruli ulcer is endemic, including Gabon, signed the Cotonou Declaration (*8*). According to this declaration, these countries have committed themselves to fight Buruli ulcer by several measures, including assessing the magnitude of the disease and conducting surveillance.
